# Construction of Customized Personas for Decision-Making Cognition Regarding Oral Microbiota Transplantation in Head and Neck Cancer Patients Undergoing Radiotherapy: A Qualitative Study

**DOI:** 10.3390/healthcare14142073

**Published:** 2026-07-10

**Authors:** Xue Liu, Hang Wang, Xinyao Yang, Yufei Li, Like Zhang, Lei Cui, Hao Li, Lili Hou

**Affiliations:** 1 School of Nursing, Shanghai Jiao Tong University, Shanghai 200025, China; xue-hang@sjtu.edu.cn (X.L.);; 2 Shanghai East Hospital, School of Medicine, Tongji University, Shanghai 200120, China; 3 Department of Endodontics, Shanghai Ninth People’s Hospital, Shanghai 200011, China; 4 Nursing Department, Shanghai Ninth People’s Hospital, Shanghai 200011, China

**Keywords:** head and neck cancer, radiotherapy, oral microbiota transplantation, decision-making, customized personas, qualitative research

## Abstract

**Highlights:**

**What are the main findings?**

- This study is among the first qualitative explorations of head and neck cancer patients’ perspectives on the emerging intervention of Oral Microbiota Transplantation for managing radiotherapy-induced complications;
- This present study proposes an innovative approach to thematic analysis, one that transcends conventional methods. This study employs a novel framework, delineating four distinct patient personas, providing a nuanced visualization of how different patients process information and approach decision-making regarding microbiota-based therapies;
- The cognitive processes involved in patient decision-making are influenced by a variety of factors, including age, education level, health literacy, psychological state, social support, and the severity of oral complications. This multifaceted environment gives rise to a range of information needs and decision-making preferences among patients.

**What are the implications of the main findings?**

- The four user personas provide a heuristic framework for healthcare providers to preliminarily identify patients’ cognitive tendencies and tailor communication strategies and educational materials according to each patient type;
- This typology establishes an empirical foundation for the future development of persona-guided decision support tools. The extent to which it can enhance decision quality, mitigate decisional conflict, or promote treatment adherence remains to be evaluated in subsequent interventional studies;
- Nevertheless, the framework provides a conceptual reference for integrating emerging microbiome therapies into radiotherapy care in a more patient-centered manner. This is achieved by accounting for heterogeneity in health literacy, psychological resilience, social support, and decision preferences.

**Abstract:**

**Background**: Patients with head and neck cancer who are undergoing radiotherapy frequently suffer from oral mucositis and oral microecological disorders, which severely impair their quality of life. Oral microbiota transplantation is an emerging oral microecological intervention that offers a novel approach for reconstructing oral microecological balance and relieving mucositis. However, regarding this innovative therapy, there is a paucity of in-depth research into patients’ decision-making cognition, and existing evidence is insufficient to support individualized clinical decision-making guidance. **Methods**: A descriptive qualitative research design was employed. From July to December 2025, patients diagnosed with head and neck cancer undergoing radiotherapy were recruited from a tertiary hospital in Shanghai via purposive sampling. The data were collected through semi-structured interviews and analyzed using Colaizzi’s seven-step analysis method. The user label system was refined and summarized to construct user portraits. These portraits were visualized in the form of WordArt word clouds and character labels. **Results**: A total of 21 eligible patients with head and neck cancer undergoing radiotherapy participated in the study. The construct of decision-making cognition encompasses five dimensions: treatment prioritization, information needs, health literacy, psychological status, and decision quality. The patients were categorized into four types: proactive participation, passive dependence, weigh carefully, and symptom-driven. These classifications reflect the cognitive characteristics and group differences regarding the Oral Microbiota Transplantation decision-making process among different patients. **Conclusions**: Patients exhibit considerable variability in their decision-making cognition regarding the innovative OMT therapy. This phenomenon can be categorized into four distinct persona types, which, respectively, reflect unique information processing styles, risk assessments, and behavioral coping strategies when patients encounter novel therapeutic interventions. This typology provides a theoretical foundation for individualized clinical decision support, delineates targets for the formulation of targeted communication strategies, and ultimately enhances patient decision quality and treatment adherence.

## 1. Introduction

Head and neck cancer is among the most prevalent diseases worldwide [1]. Radiotherapy, when utilized as the primary treatment modality [2], invariably causes irreversible damage to normal oral tissues. However, this treatment modality is effective in killing cancer cells and improving survival rates. This phenomenon can result in a series of adverse outcomes, including oral mucositis [3], xerostomia [4], and dysgeusia [5]. Radiation has been demonstrated to disrupt the balance of oral microbiology, thereby precipitating the overgrowth of pathogenic bacteria [6]. This has been shown to exacerbate oral mucosal injury and impair functions such as eating and swallowing, which severely affects patients’ quality of life [7] and may even force the interruption of radiotherapy.

Oral Microbiota Transplantation (OMT), as an emerging microecological intervention, aims to reconstruct oral microecological balance by transplanting healthy oral microbiota from donors to patients [8]. Inspiration for this approach is drawn from the principles of Fecal Microbiota Transplantation (FMT), a treatment that has been demonstrated to have notable success in the management of gastrointestinal disorders. OMT presents a novel perspective for intervening in radiation-induced oral mucositis [9].

At the preclinical stage, promising results have been observed in animal studies. Xiao et al. [9] demonstrated in a mouse model of fractionated radiotherapy mimicking head and neck cancer treatment that OMT significantly alleviated radiation-induced oral mucositis, as evidenced by reconstructed epithelium and tongue papillae, reduced leukocyte infiltration, and increased proliferative cells in the oral epithelium. Furthermore, in vivo gene silencing experiments identified S100a9 as a key mediator of OMT’s radio-protective effects, providing mechanistic support for its therapeutic potential.

In a clinical trial, Goloshchapov et al. [10] performed an oral microbiota transplantation on a 6-month-old child with neuroblastoma. The donor was the child’s healthy mother, and the transplantation material was saliva. During the transplantation cycle of OMT in this child patient, only grade 1 oral mucositis occurred, which preliminarily suggests the feasibility of OMT. In the domain of donor screening, Sonia et al. [11] have developed a novel super donor assessment tool, which aims to provide a systematic framework for OMT donor screening.

While the extant studies have demonstrated encouraging results, OMT remains in the exploratory stage. At present, there is an absence of substantial evidence concerning the most effective administration routes, dosing frequency, treatment duration, and long-term safety. Furthermore, there is a need for the establishment of unified standards for treatment protocols and efficacy evaluation [12]. In light of these substantial uncertainties, it is particularly worthwhile to investigate patients’ decision-making cognition when facing this emerging therapy.

Patients diagnosed with head and neck cancer undergoing radiotherapy make a series of decisions during the entire OMT process. At the initiation stage, patients must decide whether to undergo OMT, which involves weighing the trade-offs between this emerging therapy and conventional symptomatic treatments. During the protocol selection stage, decisions must be made regarding the route of administration and treatment timing [11]. At the implementation stage, patients must define their tolerance for risks and adverse events, including consenting to uncertainties such as the risk of potential infections. Furthermore, patients are required to participate in efficacy evaluations and subsequent treatment adjustments, determining whether the treatment needs to be modified or terminated. In conclusion, given that OMT is an emerging technology that has not yet been covered by medical insurance, patients must also make and face financial decisions. These elements of decision-making are inextricably linked to the OMT experience, forming the foundation of patients’ cognitive processes related to decision-making.

The scientific rigor of decision-making is closely linked to patient willingness and cognitive levels [13]. When confronted with OMT as a novel therapeutic modality, patients frequently encounter cognitive confusion and decisional hesitation due to a paucity of knowledge regarding the disease and treatment information [14]. The patients’ comprehension of OMT and their decisional needs, concerns and expectations directly influence their decision making and treatment adherence. However, research regarding the decision-making cognition of these patients towards OMT remains scarce internationally [15]. Consequently, the core needs and cognitive characteristics of patients during the decision-making process have not been clearly defined, nor is there sufficient evidence to support customized decision-making guidance from the patient’s perspective.

In the domain of qualitative research, Customized Personas serves as a highly effective analytical instrument. It integrates interview data to distill cognitive characteristics, decision-making preferences, and behavioral patterns across different groups. The construction of representative user profiles provides a precise, targeted basis for clinical interventions and decision-making guidance [16]. In light of these findings, the present study employs a qualitative approach to construct Customized Personas for head and neck cancer patients undergoing radiotherapy regarding OMT treatment decisions. The objective of this study is to methodically examine patients’ present cognitive status, decisional dilemmas, and influencing factors concerning OMT. Moreover, by examining patients’ psychological states, behavioral characteristics, and needs, this study aims to furnish healthcare professionals with a scientific foundation to develop effective intervention strategies.

## 2. Methods

### 2.1. Study Design and Participants

This study employed a qualitative descriptive design to explore the views of patients with head and neck cancer undergoing radiotherapy regarding the decision-making process of Oral Microbiota Transplantation. By purposive sampling, patients with head and neck cancer who received radiotherapy and were admitted to the oncology departments of tertiary hospitals in Shanghai from July to December 2025 were selected to participate in this study.

### 2.2. Inclusion and Exclusion Criteria

The inclusion criteria for patients were (1) aged between 18 and 70 years old with basic cognitive ability; (2) patients with a definite clinical and pathological diagnosis of oral and maxillofacial head and neck cancer; (3) awareness of their illness and providing informed consent; undergoing radiotherapy, understand the basic concept of “oral microbiota transplantation”; and (4) voluntarily participate in this study.

The exclusion criteria were (1) patients with mental or psychological disorders; (2) patients complicated with severe diseases of other systems, including cardiovascular, cerebrovascular, hepatic, and renal diseases; (3) patients with an expected survival time of less than 6 months; (4) clinically significant neutropenia (absolute neutrophil count < 0.5 × 10^9^/L); and (5) patients who refused to participate in this study for other reasons, such as lack of time and limited understanding of OMT.

In addition, donor screening was performed according to the established protocols of the research center.

### 2.3. Determination of Sample Size

Participants were recruited from the radiotherapy outpatient department of a tertiary hospital. This hospital has extensive clinical experience in the diagnosis and treatment of head and neck cancer and has prospectively implemented a prevention and treatment project involving Oral Microbiota Transplantation. The sample size was determined based on data saturation. Throughout the interview process, a constant comparative approach was employed. Each interview was transcribed and preliminarily coded within 24 h of completion. Two researchers independently identified newly emerging themes. Data saturation was defined as the point at which no new themes or subthemes emerged following discussion and consensus between the two coders across three consecutive interviews. To systematically document the saturation determination process, the research team maintained an audit trail that recorded in detail the emergence of new themes at each interview, coding discrepancies, and the discussion and resolution process. When no new themes emerged in three consecutive interviews, it was determined that the sample size had reached saturation, and recruitment was discontinued.

A total of 24 participants were invited to participate in the study and undergo qualitative interviews. A total of three pre-interviews were conducted to evaluate the scientific validity, rationality, and feasibility of the interview guide. However, data from these sessions were excluded from the final analysis. Consequently, 21 patients were ultimately included in the study for formal data analysis.

### 2.4. Data Collection

The objective of the sampling strategy is to collect a wide range of viewpoints from diverse perspectives. The strategy aims to maximize variations in age, educational level, tumor location and pathological type. These factors are believed to potentially influence patients’ decision-making process regarding OMT. Following the acquisition of informed consent, the subjects’ general demographic data was collected. This included gender, age, educational level, marital status, work status, place of residence, type of head and neck cancer, radiotherapy cycle, and whether they participated in OMT. At that time, none of the participants had actually received OMT in clinical practice.

Semi-structured, face-to-face, one-on-one interviews were conducted by the first author, a master’s student who has completed the qualitative research course. In an effort to mitigate potential interviewer bias and social desirability effects, the following measures were implemented. First, participants were recruited independently through the radiotherapy outpatient clinic, and the first author had no prior clinical or personal relationship with any participant before the interview. Second, prior to each interview, the participants were explicitly informed that (a) participation was entirely voluntary; (b) there were no right or wrong answers; (c) their responses would not affect their medical care in any way; and (d) they could withdraw at any time without consequence. Third, interviews were conducted in a quiet room within the radiotherapy department or the hospital’s interview room to ensure a quiet and comfortable environment, free from interruptions or the presence of clinical staff. The interview is recorded in its entirety through the use of audio equipment. During face-to-face interviews, the interviewee’s expressions, emotions and other nonverbal behaviors are meticulously observed and documented. Each interview lasts for 20 to 30 min. Subsequent to each interview, the primary author is responsible for transcribing and arranging the audio materials within 24 h. The interviewer engaged in reflexive thinking throughout the study, taking brief notes after each interview on how her role might have influenced participant responses. These reflexive notes were discussed within the research team to contribute to methodological awareness. To ensure the trustworthiness of the analysis and the risk of individual researcher bias, a dual-coding approach was adopted. Two researchers independently coded the data and compared the results. Discrepancies in coding were resolved through discussion within the research team. If no consensus was reached, a third senior researcher was consulted. This separation of data collection and data analysis roles ensured that the final thematic interpretations were not based on individual judgment.

### 2.5. Interview Guide

Based on the research objectives, a preliminary interview guide was drafted through a comprehensive review of the domestic and international literature and discussions with the research team. Subsequently, expert consultation was conducted to finalize the interview guide. We conducted a pre-interview with 3 patients who met the inclusion criteria. Before the pre-interview, we explained the purpose and content of the study to the respondents again and began the interview after obtaining their consent. Based on the pre-interview results, we refined the clarity and relevance of the interview guide and the relevance and operability of the interview outline and, finally, determined the formal interview outline as follows: (1) How did you first learn about oral microbiota transplantation (OMT)? What do you think OMT is, and what do you think needs to be done for OMT treatment? What are the potential risks and benefits of OMT treatment in your opinion? (2) Has anyone (such as doctors, nurses, family members or friends) ever provided you with support and assistance when you were considering whether to receive OMT treatment? What conditions or information can help you make the treatment decision more quickly? (3) When making a decision on OMT treatment, what is the first factor you consider? What would you do if your family members or medical staff had different opinions on your decision? (4) What thoughts and feelings do you have during the process of understanding and considering OMT treatment? Are you satisfied with the decision-making process you have experienced so far? (5) What kind of support or information do you expect to assist you in the OMT treatment decision-making process? What help, guidance or information support do you hope to receive in the process of making OMT treatment decisions and subsequent treatment?

### 2.6. Data Analysis

#### 2.6.1. Establish the Dimension of Character Portraits

The construction process of a character portrait is typically comprised of three steps: data collection, feature extraction, and portrait representation. In this study, Colaizzi’s seven-step analysis method was adopted with the aid of NVivo 15.0 software to analyze and preprocess the collected text data. The analysis process involves several steps, including familiarization with the text, identification of meaningful statements, construction of code, clustering of topics, provision of detailed descriptions, generation of basic structures, and verification.

The analysis and processing process involved two researchers who independently read the interview data, extracted relevant statements and formed corresponding codes. Subsequently, the two researchers collaborated to review the codes, with the objective of identifying the types and topics. These elements were integrated as the common characteristics of the participants. These common characteristics form the basic dimensions of the patient role label, which are used to describe the characteristics, cognitive level and decision-making needs of the participants. In instances where the results of the two researchers’ analyses differed, the research team convened to reach a consensus, with the objective of ensuring the scientific rigor and accuracy of the code extraction and topic clustering.

#### 2.6.2. Construct Character Portraits

In this study, the features of the character portraits were extracted manually. The research team conducted a collaborative analysis of the characters’ features across various dimensions. This analysis was informed by three sources: the dimensions of the character portraits, the transcribed text data from interviews, and clinical experience. Participants with analogous characteristics and needs were grouped to construct their group personas.

To ensure the credibility and accuracy of the constructed character portraits, a member validation process was undertaken. The specific procedure was as follows. The preliminary character portraits (descriptive narratives, key characteristics) were synthesized into a structured document. These portraits were subsequently presented face-to-face to the participants assigned to each corresponding character group. Participants were invited to evaluate whether (a) the portrait features accurately reflected their personal experiences; (b) the coded content faithfully captured their own thoughts; and (c) any aspects had been omitted, overemphasized, or misinterpreted.

After a thorough review by the research team, it was determined that the constructed characters exhibited a high degree of accuracy in reflecting their traits. This method ensures the accuracy and detail of the constructed character roles and represents the different characteristics of the study population.

#### 2.6.3. Expression of Character Portraits

**Figure 1:**
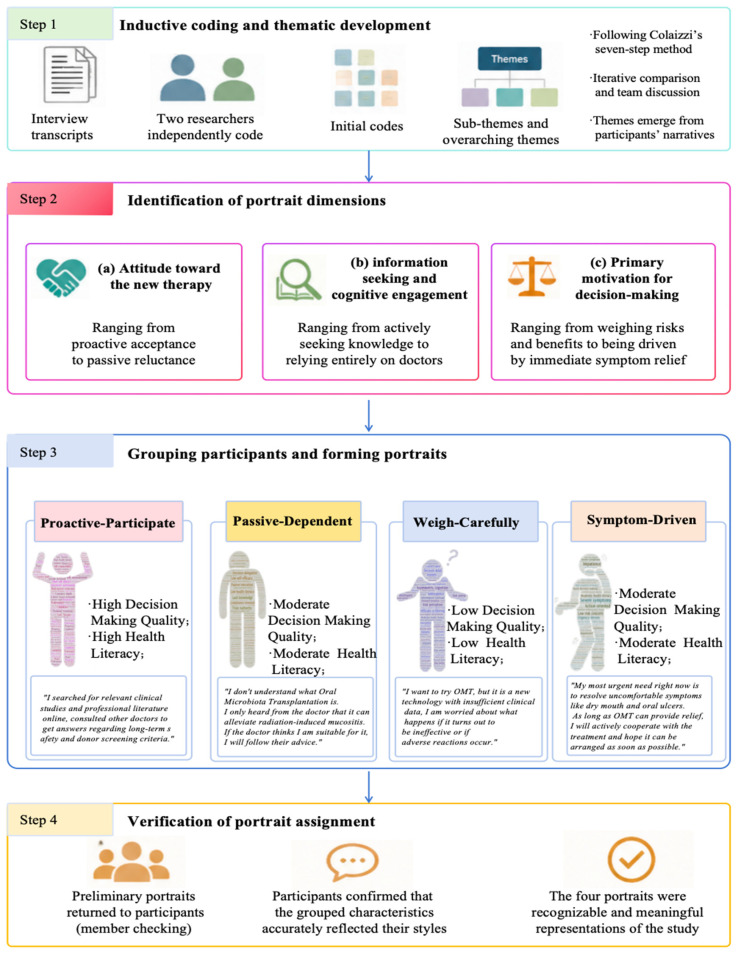
Development of patient decision-style portraits through inductive coding and thematic analysis.

Portrait representation is defined as the process of visually presenting groups with distinct characteristics. There are several common methods for constructing character portraits. These include word cloud diagrams, trait-based character charts, and statistical charts. In this study, WordArt v4.34 was used to generate word cloud maps, which were used to visually represent the character portraits of patients with head and neck cancer undergoing radiotherapy. These maps were used to assess the patients’ cognition of OMT treatment decision-making. Among them, the higher the frequency of label appearance in the word cloud was positively correlated with the label font size. This suggests that the participants’ core cognitive points and focuses of attention. These visual representations of the character portraits offer a clear illustration of the diversity in cognition and decision-making preferences regarding OMT treatment among head and neck cancer patients undergoing radiotherapy. Detailed steps in Figure 1.

### 2.7. Rigor

In this study, both the data collectors and analysts have received professional qualitative research training and are competent in conducting qualitative research. To ensure the quality of the interviews, the researchers made proper arrangements for the interview time and location in advance. They also clarified the value and significance of the research to the respondents and gained their trust. Throughout the interviews, they remained strictly neutral and refrained from making any suggestive or guiding remarks. It is imperative to acknowledge the role of reflexive practices in enhancing the credibility of survey results. Such practices encompass the documentation of potential biases during the research process and the facilitation of regular team discussions.

### 2.8. Ethical Considerations

Ethical approval was obtained from the Institutional Review Board of The Ninth People’s Hospital Affiliated to Shanghai Jiao Tong University School of Medicine [Approval No.: SH9H-2024-T437-2]. All participants read the study’s purpose and were assured of their right to withdraw at any time without affecting their care. Written informed consent was obtained prior to interviews. All data were anonymized and stored securely on a password-protected standalone hard disk accessible only to the research team.

## 3. Results

### 3.1. Basic Information of Participants

This study included 21 patients with head and neck cancer undergoing radiotherapy (Supplementary File S1). Among them, there were 8 males and 13 females, with ages ranging from 37 to 68 years (mean age: 52.76 years). Regarding educational attainment, 66.67% of the participants had obtained a middle education or higher. Furthermore, 85.71% were married, 47.62% were employed, and 71.43% resided in city areas. The cancer sites included the tongue, parotid and others. Regarding pathological types, 52.38% were squamous cell carcinoma (SCC), and 28.57% were adenoid cystic carcinoma (ACC), as shown in Table 1. All participants were informed of the study details and provided consent to participate.

### 3.2. Customized Personas for Decision-Making Regarding Oral Microbiota Transplantation in Patients with Head and Neck Cancer Undergoing Radiotherapy

Based on the qualitative analysis, this study classified patients with head and neck cancer undergoing radiotherapy into distinct groups according to their decision-making cognition regarding OMT treatment. Among the 21 participants who completed the formal interviews, 15 (71.4%) agreed to take part in the verification process and provide feedback. Among them, 12 (80.0%) fully agreed with the role classification they were assigned to, confirming that the portrait accurately reflected their experiences and decision-making processes. The remaining three (20.0%) expressed partial agreement, indicating the degree of concern or specific reasons for hesitation regarding the side effects of OMT, which could be further clarified. Based on this feedback, the research team reviewed and revised the statements of the corresponding role groups. A total of four cognitive profiles were constructed with different head and neck cancer patients, namely the Proactive–Participate type, the Passive–Dependent type, the Weigh-Carefully type, and the Symptom-Driven type. The characteristics and decision-making needs of each profile are detailed in Table 2.

Theme 1: Decision-making Cognition regarding OMT among Proactive-Participating Patients

Patients in this category were relatively young (aged 35–55), predominantly urban residents with higher educational attainment. They actively engaged in the OMT decision-making process by taking the initiative to understand the mechanism, potential risks, and benefits of OMT. When encountering questions during decision-making, they proactively consulted healthcare providers, family members, friends, and professional channels to seek support and information. Consequently, their decision-making was of relatively high quality.

P3: “*I searched for relevant clinical studies and professional literature online, consulted other doctors to get answers regarding long-term safety and donor screening criteria. The doctor mentioned that my questions were very professional.*”

P8: “*The doctor explained the specific procedures and potential adverse reactions of OMT in detail. I believe this information is of significant value for decision-making, and I will discuss it with relevant parties. I will discuss this with my family to make a rational decision, and if I decide to undergo OMT, I will strictly comply with the doctor’s instructions.*”

P21: “*I joined a patient support group to share my experiences with OMT treatment. When I felt confused, I could draw on the experiences of other patients and learn a great deal of useful dietary precautions.*”

P16: “*My job is related to the healthcare industry, so I am familiar with Fecal Microbiota Transplantation and know it has good outcomes. This is my first time hearing about Oral Microbiota Transplantation, and I hope to give it a try.*”

P6: “*Ever since I learned about the importance of oral microecology, I have taken the initiative to purchase probiotic yogurt and related products, hoping they will help alleviate my mucositis.*”

Theme 2: Decision-making Cognition regarding OMT among Passive–Dependent Patients

Patients in this category were generally elderly, aged between 55 and 75, and had moderate educational backgrounds. While they possessed a basic understanding of OMT’s role in alleviating oral symptoms, they had limited awareness of its potential risks and technical details. When making decisions about OMT, they tended to rely heavily on healthcare providers and family members and often struggled to independently address their questions or concerns. Consequently, their decision-making quality was considered moderate.

P5: “*I don’t understand what Oral Microbiota Transplantation is. I only heard from the doctor that it can alleviate radiation-induced mucositis. If the doctor thinks I am suitable for it, I will follow their advice.*”

P18: “*My son helped me look up a great deal of information. I’m not very good with the internet myself, and those complicated principles go right over my head.*”

P11: “*I have always been worried about the side effects of OMT. The doctor told me to pay attention to oral hygiene after treatment, and nurses taught me step-by-step how to rinse my mouth and perform oral care. Without their guidance, I really wouldn’t know what to do.*”

Theme 3: Decision-making Cognition regarding OMT among Weigh-Carefully Patients

Patients in this category were primarily aged 45–65, residing in urban centers and suburbs, with moderate educational and economic backgrounds. While they possessed a certain understanding of the efficacy and safety of OMT, their core focus remained on potential risks and uncertainties. They tended to be indecisive and prone to anxiety during the decision-making process. With only an average ability to screen information and make judgments, and given the limited information available, they require additional clinical evidence and support to reach a definitive conclusion. Consequently, their decision-making quality was deemed moderate-to-low.

P7: “*I want to try OMT, but since it is a new technology with insufficient clinical data, I am worried about what happens if it turns out to be ineffective or if adverse reactions occur.*”

P2: “*I heard that OMT involves transplanting microbiota from others. Can the donor’s health be guaranteed? Is there a risk of infectious diseases? My family and I are quite hesitant.*”

P19: “*I’m weighed down by immense mental pressure, and I’m especially terrified that any missteps in the procedure could hinder my recovery. I also fear that making the wrong decision will impact my subsequent treatment plans.*”

P20: “*I saw mixed reviews online regarding microbiota transplantation, including some negative cases. The more I read, the more worried I became, unsure if OMT is actually reliable. I hope doctors can explain the adverse reactions in detail so that I can have more confidence in my decision.*”

Theme 4: Decision-making Cognition regarding OMT among Symptom-Driven Patients

Patients in this category were aged 40–60, covering middle-aged and older adults, and primarily resided in urban centers. They suffered from severe oral complications post-radiotherapy, such as mucositis, xerostomia, and pain. While they had a limited understanding of the technical principles and risks of OMT, their core decision-making driver was the relief of oral discomfort. Driven by a strong sense of urgency, they paid little attention to technical details and long-term outcomes, demonstrating a clear and intense willingness to receive treatment. Consequently, their decision-making quality was largely influenced by the severity of their symptoms.

P4: “*I experienced severe pain in my mouth after radiotherapy, which strictly limited my eating and drinking. As long as this Oral Microbiota Transplantation can alleviate my pain and restore my basic eating function, I am willing to accept the treatment.*”

P12: “*My most urgent need right now is to resolve uncomfortable symptoms like dry mouth and oral ulcers. As long as OMT can provide relief, I will actively cooperate with the treatment and hope it can be arranged as soon as possible.*”

P18: “*If it can rapidly alleviate my oral symptoms, I don’t mind paying a bit more. You don’t need to explain too much professional knowledge to me; just tell me how to treat it and when it can be done.*”

## 4. Discussion

### 4.1. Diversity in Decision-Making Cognition Regarding OMT Among Head and Neck Cancer Patients Undergoing Radiotherapy

Patients exhibit significant variation across a range of demographic characteristics, educational levels, and severity of oral mucositis [17]. Our sample included patients with different head and neck cancer subtypes, primarily ACC (28%) and SCC (52%). It is noteworthy that there was an absence of ACC patients in the active participation category. The group consisted of four SCC patients and one patient with other pathological types. In contrast, ACC patients were distributed across the remaining three types. The hypothesis is proposed that the pathological type of the tumor may be a contributing factor to patients’ cognition of OMT decision-making. However, the limited sample size precludes the possibility of conducting a formal subgroup analysis by tumor type. Subsequent studies with larger sample sizes will be necessary to validate the stability of these classifications across different subtypes of head and neck cancer.

The cognitive processes involved in decision-making are influenced by a combination of intrinsic factors, such as health literacy and psychological resilience [18], as well as extrinsic factors, including social support and information accessibility [19]. In contrast to conventional treatments that are guided by established guidelines, OMT remains in the nascent stages of clinical application. Consequently, patients’ decision-making cognition regarding this emerging biotherapy exhibits significant diversity, which can be categorized into four core types: Proactive–Participate, Passive–Dependent, Weigh-Carefully, and Symptom-Driven.

However, this typology is derived from an exploratory qualitative analysis, rather than fixed patient categories. Consequently, individual patients may exhibit overlapping traits across different consultation stages. Therefore, rather than prescribing a uniform strategy, clinicians may use this framework as a heuristic reference to preliminarily identify patients’ cognitive tendencies [20].

### 4.2. Proactive–Participate Patients

The demographic composition of this group under scrutiny is predominantly characterized by its middle and young age structure, urban residence and highly educated. With regard to information acquisition, they exhibit a notable degree of initiative. By searching for authoritative data, consulting healthcare providers and joining patient communities were undertaken to ascertain information regarding the long-term safety of OMT, the donor screening criteria, and the mechanisms of action, This approach reflects a high level of health self-efficacy [21]. Simultaneously, they exhibit remarkable psychological resilience. When facing the uncertainties of emerging therapies, they maintain calm and rational judgment, alleviating anxiety through active learning and information integration [22]. Furthermore, they have also exhibited a propensity to encourage family members to engage in the decision-making process, underscoring a collaborative approach to healthcare decision-making. In terms of social support, they facilitate efficient communication with medical teams and families. This enables the prompt acquisition of professional guidance, the verification of information accuracy, and the optimization of decision-making through peer exchange. Notably, some patients in this category possess relevant medical backgrounds, enabling them to draw analogies between the relatively mature Fecal Microbiota Transplantation and OMT to reduce cognitive uncertainty. This finding suggests that prior domain knowledge can offer targets for the acceptance and understanding of new technologies and therapies [23].

For this patient subgroup, the emphasis of the consultation process may undergo a shift from the dissemination of information to the validation of information and the collaborative analysis of data. Clinicians can engage these patients by providing clarification on ambiguities present in self-obtained data and by engaging in a collaborative interpretation of the available risk–benefit evidence. With regard to family involvement, healthcare professionals are encouraged to elicit critical questions from patients and their families during consultations. This approach is intended to ensure that patients receive comprehensive information and support, while also respecting their autonomy. Regarding decision support tools, providing the latest clinical trial summaries is of paramount importance. Ultimately, this approach is predicated on the objective of safeguarding their autonomy while anchoring their independent exploration in professional evidence, necessitating further investigation in future studies.

Across the four dimensions, this subgroup demonstrates high health literacy, robust psychological resilience, and social support that operates as a co-analysis rather than surrogate decision-making. Their decision-making process is characterized by a preference for a symmetrical dialogue with clinicians, where they seek validation for self-obtained evidence. Consequently, customized assistance may prioritize interactive data platforms over fundamental education, enabling clinicians to allocate their limited time to rectifying misinterpretations.

### 4.3. Passive–Dependent Patients

The demographic under scrutiny is predominantly composed of older adults who have attained relatively limited levels of education, exhibiting behaviors and characteristics that are analogous to those observed in individuals diagnosed with dependent personality disorder. In the decision-making process, significant reliance is placed on healthcare providers and family members to gather and organize information, as the subjects frequently encounter challenges in independently addressing issues and exhibit a deficiency in self-assurance regarding their decisions and actions. These subjects demonstrate a high degree of compliance but low autonomy. Confronted with OMT decisions, individuals may experience feelings of fear and helplessness, leading to a deficiency in the confidence necessary for independent judgment. Consequently, they have a propensity to adhere to professional counsel, assuming the role of executors rather than contributors to the decision-making process. This phenomenon aligns with extant research on surrogate decision-making in oncology, where elderly patients frequently delegate decision-making authority to trusted individuals [24]. Nevertheless, this study further reveals that such dependence is not exclusively attributable to declining cognitive abilities but is predominantly associated with inadequate health literacy [18]. Seo et al. [25] found that the proportion of individuals with adequate health literacy who participated in decision-making was twice that of those with limited health literacy. As the internet becomes the primary source of health information, this issue is becoming increasingly prominent [26].

For this patient subgroup, communication should prioritize cognitive load reduction. Clinicians can adopt simplified and visual communication strategies [27], such as the use of illustrated brochures, verbal explanations, and on-site demonstrations. In terms of family involvement, relatives may receive brief training in bedside care, including the translation of medical jargon into daily oral care instructions and medication reminders.

Across the four dimensions, this subgroup exhibits low health literacy and low decision-related self-efficacy, with social support assuming surrogate decision-making functions. Their inclination is toward the provision of directive recommendations. From a clinical perspective, the implementation of audio–visual step-by-step guides and family caregiver training has the potential to mitigate cognitive burden while progressively enhancing autonomous capacity. This outcome necessitates longitudinal observation to ensure its assessment.

### 4.4. Weigh-Carefully Patients

The group under consideration is predominantly composed of middle-aged adults experiencing typical decisional conflict, a condition that arises when individuals weigh risks and benefits in the context of uncertainty. This condition is often accompanied by distress and decision delay [28]. While these patients possess a fundamental understanding of OMT and understand its therapeutic benefits, they demonstrate an insufficient capacity to process and evaluate information. Consequently, they are easily disturbed by negative cases and fragmented online information, leading to excessive worry about potential risks. This phenomenon is receiving increasing attention in the context of precision medicine and emerging biotherapies [29]. Despite the support of family members and healthcare providers, these patients continue to experience anxiety during the decision-making process and face challenges in mitigating their exaggerated perception of risk.

For this group, clinical staff must prioritize precise communication. The mere provision of clinical case data and long-term efficacy follow-up results is insufficient. Rather, the provision of targeted decision support tools is necessary. Research by Ozanne et al. [30] demonstrated that decision aids utilizing visual analogies and probability estimation can reduce patient decisional conflict. Concurrently, the organization of collaborative forums with patients who have effectively experienced the benefits of OMT can serve as a counterbalance to the influence of negative online information [31]. Furthermore, the establishment of a multidisciplinary decision support team that integrates expert opinions from oncology, infectious diseases, and rehabilitation can provide comprehensive advice, gradually enhancing their confidence in decision-making.

Across the four dimensions, this subgroup is distinguished by moderate health literacy coupled with high anxiety, where social support paradoxically introduces competing opinions that amplify decisional conflict. Their inclination is toward exhaustive deliberation. However, they demonstrate an absence of confidence in synthesis. The strategies previously delineated—visual analogies, peer sharing, and multidisciplinary teams—directly address these specific barriers; their comparative effectiveness warrants further study.

### 4.5. Symptom-Driven Patients

This group consists primarily of older adults who have experienced severe complications following radiotherapy, such as oral mucositis, xerostomia, and pain. For these patients, decision-making is symptom-driven [32], a phenomenon also evidenced in acute symptom contexts such as pain management [33] and palliative care [34]. Driven by the urgent need to alleviate physical suffering, they pay less attention to long-term risks and technical details, focusing instead on practical information such as rapid pain relief and the restoration of eating functions. The decisions of these individuals are predominantly influenced by their perception of symptoms, with recommendations from family members and healthcare professionals serving as supplementary references. Their inclination to make a decision is characterized by its directness and intensity [35]. This aligns with Maslow’s hierarchy of needs in symptom management, where the satisfaction of physiological needs takes precedence over higher cognitive interventions [36]. For these patients, clinical staff should aim to reduce symptom severity through rapid intervention [37]. The primary objective is to elucidate the OMT treatment process and the essentials of daily care, while concurrently formulating personalized treatment plans. Once symptoms are initially controlled, patients should be gradually guided to focus on long-term management and risk prevention, facilitating a dynamic transformation from a symptom-driven to a proactive-participating pattern.

For this subgroup, health literacy and psychological engagement are temporarily superseded by acute physical distress. Social support is transformed into instrumental assistance—symptom recording and care coordination. Clinicians have demonstrated a preference for immediate and practical approaches. They have been known to initiate treatment with rapid symptom assessment tools and to subsequently introduce more comprehensive decision-making content as symptoms stabilize.

## 5. Conclusions

Through in-depth interviews with 21 head and neck cancer patients undergoing radiotherapy, this study constructed four cognitive types of decision-making regarding OMT: Proactive–Participate, Passive–Dependent, Weigh-Carefully, and Symptom-Driven.

The primary cause of patient anxiety and decisional hesitation is among Proactive–Participate patients. The distinction between Proactive–Participate and Passive–Dependent patients is predicated on disparities in educational attainment and digital health literacy. Family and peer support exhibit a multifaceted role, encompassing aspects such as information empowerment and surrogate decision-making, which vary across typologies. This heterogeneity is indicative of variances in health literacy, psychological resilience, social support, and decision preferences. This typology is intended to serve as an exploratory framework, with the objective of identifying targets for the implementation of personalized clinical decision support. It is designed to facilitate the establishment of rational and balanced risk perceptions among patients, with the ultimate aim of enhancing their decision-making confidence. It is imperative to provide a heuristic reference for tailoring communication strategies and the development of targeted decision aids. In light of the ongoing advancements in OMT and the analogous microbiome intervention technologies, a profound comprehension of the heterogeneity in patient decision-making cognition is imperative for effectively translating medical innovations into patient-centered clinical practice.

## 6. Limitations

This study has several limitations. First, the findings are derived from a small qualitative sample of 21 patients recruited from a single tertiary hospital in Shanghai, with urban residents accounting for 71.4%. Consequently, the distribution patterns of the four cognitive types should be interpreted with caution, and the results remain exploratory and context-specific rather than generalizable. Furthermore, the inclusion criterion requiring basic awareness of OMT may have excluded patients with no prior knowledge of this therapy, potentially resulting in an under-representation of the full spectrum of head and neck cancer patients. To validate these classifications, larger-scale quantitative studies are required, encompassing a broader geographic range, rural populations, and primary-care settings. Secondly, as a descriptive qualitative study, although strategies such as Colaizzi’s seven-step analysis, team discussions, and participant verification were employed to control subjectivity, the construction of patient typologies inevitably carries interpretative components from the researchers. Future mixed-method designs could further explore the evolution of these decision-making patterns throughout the OMT treatment trajectory. Thirdly, the participants were exclusively from China. The interviews were conducted in Chinese, while the data analysis and manuscript writing were performed in English. This may introduce potential bias in translation and interpretation. Despite these limitations, the typology offers a heuristic framework for understanding patient heterogeneity in OMT decision-making and provides empirically grounded hypotheses for future investigation.

## Figures and Tables

**Table 1:** General information about participants.

Variable		Frequency (*n*)	Percentage (%)
Age (years)	18~44	3	14.29
	45~59	12	57.14
	>60	6	28.57
Gender	Female	13	61.90
	Male	8	38.10
Educational level	Primary	7	33.33
	Middle	6	28.57
	University	8	38.10
Marital status	Married	18	85.71
	Unmarried	2	9.52
	Divorced	1	4.76
Employment status	Employed	10	47.62
	Farmer	6	28.57
	Retired	5	23.81
Residential address	City	15	71.43
	Village	6	28.57
Tumor site	Tongue	8	38.10
	Parotid	3	14.29
	Gums	3	14.29
	Neck	2	9.52
	Scalp	1	4.76
	Skull	1	4.76
	Palatine	1	4.76
	Others	2	9.52
Pathological type	SCC	11	52.38
	ACC	6	28.57
	Other	4	19.05

**Table 2:** Characteristics and decision-making priorities of the four customized patient personas regarding Oral Microbiota Transplantation.

Name	Proactive–Participate Patients	Passive–Dependent Patients	Weigh-Carefully Patients	Symptom-Driven Patients
Individuals	P3, P8, P16, P21, P6	P5, P11, P17, P9, P18	P7, P2, P19, P10, P4, P20	P12, P13, P14, P15, P1
Gender and age	2 Male and 3 Female Aged 38~52	2 Male and 3 Female Aged 55~68	2 Male and 4 Female Aged 37~63	2 Male and 3 Female Aged 42~60
Educational Level	University: 4 people Middle: 1 person	Primary: 3 people Middle: 2 people	Primary: 3 people Middle: 1 person University: 2 people	Primary: 1 person Middle: 2 people University: 2 people
Employment status	Employed: 5 people	Farmer: 3 people Retired: 2 people	Employed: 3 people Farmer: 1 person Retired: 2 people	Employed: 2 people Farmer: 2 people Retired: 1 person
Residential address	City: 5 people	City: 2 people Village: 3 people	City: 5 people Village: 1 person	City: 3 people Village: 2 people
Pathological type	4 SCC and 1 Other	2 SCC and 3 ACC	3 SCC, 2 ACC and 1 Other	2 SCC, 1 ACC and 2 Other
Treatment Priority	symptomatic relief = security	Medical advice > symptomatic relief	security > symptomatic relief	symptomatic relief > security
Information Requirements	High-quality clinical evidence, efficacy data, and long-term safety information.	Clear and simple guidance; assistance from family members to obtain information.	Comprehensive risk benefit communication; clinical evidence and success rate data;	Quick access to treatment; clarify the treatment process and time arrangement.
Health Literacy	High	Moderate	Low	Moderate
Psychological State	Confident, proactive and in control; believe the ability to be responsible for treatment decisions	Feel at ease when following doctor’s advice; anxiety when making independent decisions	Anxiety, ambivalence and uncertainty; fear of making wrong decisions	Eager to relieve symptoms; psychological focus on pain and discomfort.
Decision Making Quality	High	Moderate	Low	Moderate

## Data Availability

The original contributions presented in this study are included in the article/Supplementary Material. Further inquiries can be directed to the corresponding author.

## Supplementary Materials

The following supporting information can be downloaded at https://www.mdpi.com/article/10.3390/healthcare14142073/s1, File S1: Demographic and clinical characteristics of each participants.

## Abbreviations

The following abbreviations are used in this manuscript:

OMT	Oral Microbiota Transplantation
FMT	Fecal Microbiota Transplantation
SCC	Squamous Cell Carcinoma
ACC	Adenoid Cystic Carcinoma
